# Psychiatric Diseases in Gastrointestinal Cancer Survivors: Prevalence and Impact on Overall Survival

**DOI:** 10.1192/j.eurpsy.2026.10652

**Published:** 2026-07-31

**Authors:** F. Mulita, E. Shaska, E. Liolis, L. Tchabashvili, K. Tasios, P. Leventis, N. Kornaros, A. Antzoulas, D. Litsas, G.-I. Verras

**Affiliations:** 1 Department of Surgery, University of Patras, Patras; 2 Department of Surgery, General Hospital of Aigio, Aigio, Greece; 3 Head of Department of Emergency Psychiatry, “Ali Mihali” Psychiatric Hospital, Vlora, Albania; 4 Department of Oncology, University of Patras, Patras; 5 Department of Surgery, General Hospital of Lamia, Lamia, Greece

## Abstract

**Introduction:**

Gastrointestinal (GI) cancers, including stomach, liver, esophageal, pancreatic, and colorectal cancers, represent more than 26% of all malignancies. Colorectal cancer (CRC) represents the third most frequently diagnosed malignancy among both men and women and stands as the second leading cause of cancer-related mortality globally. In 2020, the International Agency for Research on Cancer (IARC) estimated that there were approximately 2 million new cases of CRC and 1 million CRC-related deaths globally. These figures accounted for 10% of the worldwide cancer incidence and 9.4% of all cancer-attributable mortality. Many cancer survivors, including those with colorectal cancer (CRC), commonly experience physical and mental health problems that are typically sequelae of the malignancy and its associated treatments. A cancer diagnosis is frequently intertwined with the presence of mental illness and comorbid health conditions and patients with pre-existing mental illness, in particular, may face a worse overall prognosis, potentially leading to increased mortality. The incidence of new-onset mental health conditions in GI patients is particularly elevated among those with additional medical comorbidities, post-cancer complications, or sequelae directly attributable to the surgical intervention

**Objectives:**

Ιn this prospective single-center study, we involved 603 patients undergoing stomach, liver, esophageal, pancreatic, or colorectal cancer operations at the Department of General Surgery of the General University Hospital of Patras between January 2019 and December 2023. This research focused on mental diseases as long-term complication that appeared in these patients one year postoperatively.

**Methods:**

Psychological and psychiatric support was offered as part of routine practice within the standard patient follow-up and multidisciplinary oncological treatment framework. Consultant psychiatrists performed psychiatric workups and assessments in outpatient clinics. Patients were followed for one year following their GI treatment within the scope of this study.

**Results:**

Of the 603 patients, 126 (20.9%) developed at least one psychiatric diagnosis during the follow-up period. The most frequently observed conditions were mood disorders (20.6%), primarily depressive disorders (19%), and anxiety disorders (12.7%), followed by delirium and other cognitive disturbances (19.5%).

**Conclusions:**

In conclusion, mental health disorders constitute a pivotal yet insufficiently acknowledged aspect of postoperative care for patients with GI cancer, especially among those experiencing complications. Recognizing mental health as a fundamental element of oncological patient care, supported by existing evidence of its influence on overall survival, is paramount.

**Disclosure of Interest:**

None Declared

